# Hereditary Syphilis

**Published:** 1873-12

**Authors:** 


### ARTICLE X.

### *Hereditary Syphilis.*

I saw, in Prof. Jones's office, a skeleton of a female who died of hereditary syphilis. I will copy Dr. Jones's description of the case, as it appears in his pamphlet entitled *Clinical Memoranda :*

" I examined this unfortunate woman in the Charity Hospital only a day or two before her death. At the time of my observation she appeared to be exceedingly feeble, and was a mass of offensive running sores. I obtained the body after death, and had the skeleton carefully prepared. The hymen was perfect, and the disease appeared to have been derived from inheritance, and to have manifested itself from early childhood. The feet was very small, not much larger than those of a child four years old, and appear never to have been used in walking. All the long bones of the body were more or less carious, and, in almost every case, were punctured or ulcerated through, at one or more places. The pelvic bones were carious, and the os sacrum was a mere shell. The vertebræ, were all carious. The upper jaw contained one small tooth, and the lower jaw three teeth. The alveola were absorbed. The outer and inner tables of the skull were perforated in several different places. The lower jaw, on the right side, was eroded through. The position of those fractures and erosions were marked during life, by open running sores.

"It is impossible with the pen, to portray, adequately, the terrible condition of this unfortunate female, whose bones literally rotted, piecemeal by piecemeal, during life. And the best description which we can give of the skeleton is, to say the bones of the feet, ankle, os calis, were carious ; the tibia and fibula, the femur, the pelvic bones, the os sacrum' the radius, ulna, the humerus, the scapulæ and sacrum, the lower jaw and cranium, were all eroded through in various places."

This case I regard as one of the most remarkable on record, and has been very accurately described by Professor Jones ; it clearly shows the tendency of constitutional syphilis to undermine and break down the constitution, sooner or later, if not eradicated from the organism, which may be possible at a given stage, notwithstanding the immense weight of testimony to the contrary.

We discover, in this case, the great tendency of this disease to locate itself in the bones, from some strong affinity the lime of the bones has for the specific virus, which, we naturally conclude, is of an acid character. Notwithstanding it is ascertained that the disease produces a profound change in every cell and corpuscle of every tissue of the body, until it is permanently seated in the bones; there may be some chance for permanent eradication, if properly understood and treated in time.

One striking peculiarity I will mention in relation to this case. The three teeth in the lower jaw, the left cuspid and the right central and lateral incisors, are large, and well developed in every respect; though the alveolæ is extensively absorbed, the teeth are firm in the bones. There are no grooves, pits, or notches, as might have been predicted. These teeth are the only exception to the ravages of the disease. The upper tooth I did not see. There are no evidences in the jaw bones of her ever having had any more teeth, though it is presumable that she did, and that she lost them by alveolar absorption, as the three teeth show no evidence of decay. The mouth, at least during the period of second dentition, must have been comparatively healthy, and free from the disease, from the indications of the three teeth.

In the Museum of the Medical Department of the University of Louisiana, Professor Joseph Jones has a collection of skeletons, implements of warfare, images, vases of religious worship, besides cooking utensils of various kinds, from the Mounds of Middle Tennessee. These are the remains of the mound-builders, whose history, with their barbarous civilization, is entirely lost; conjecture alone remains. I noticed about a dozen skulls, and most of the other bones belonging to them.

In that collection of bones, I notice evident signs of constitutional syphilis, either hereditary or induced—most likely the former. There were nine tibia, and five fibula, slhowing unmistakable evidence of the disease; the spines of the tibia were quite extensively corroded, mostly the entire length.

I did not find any evidence of the disease about the skulls; the teeth, in particular, were perfectly natural and sound—no furrowing or pitting of enamel, which is sometimes noticed in hereditary disease, or even induced during infancy.

These facts show conclusively that syphilis is not altogether a creature of civilization.

Another curious case of furrowed enamel was that of a lad about 14 years old, who said he had the measles when five or six years old, and was not expected to live for a long time. The two upper central incisors were grooved across, near their cutting edges or points. The four six-year molars were similarly grooved all around the borders of the grinding surfaces, with sharp points projecting up at each cusp. The father and elder brother, also the lad himself, stated that the grooving was up close to the gums after the teeth were fully grown, and that the grooving continued to grow off, or towards their points, and was perceptible from year to year.

The teeth were all well developed, and sound.—*Prof. Cutler on Furrowed Enamel.—Nashville Journal of Med. and Surgery.*

